# LC-PUFA-Enriched Oil Production by Microalgae: Accumulation of Lipid and Triacylglycerols Containing *n*-3 LC-PUFA Is Triggered by Nitrogen Limitation and Inorganic Carbon Availability in the Marine Haptophyte *Pavlova lutheri*

**DOI:** 10.3390/md11114246

**Published:** 2013-10-30

**Authors:** Freddy Guihéneuf, Dagmar B. Stengel

**Affiliations:** Botany and Plant Science, School of Natural Sciences, Ryan Institute for Environmental, Marine and Energy Research, National University of Ireland Galway, Galway, Ireland; E-Mail: dagmar.stengel@nuigalway.ie

**Keywords:** microalgae, sodium bicarbonate, triacylglycerol, *n*-3 LC-PUFA, *Pavlova lutheri*

## Abstract

In most microalgal species, triacyglycerols (TAG) contain mostly saturated and monounsaturated fatty acids, rather than PUFA, while PUFA-enriched oil is the form most desirable for dietary intake. The ability of some species to produce LC-PUFA-enriched oil is currently of specific interest. In this work, we investigated the role of sodium bicarbonate availability on lipid accumulation and *n*-3 LC-PUFA partitioning into TAG during batch cultivation of *Pavlova lutheri*. Maximum growth and nitrate uptake exhibit an optimum concentration and threshold tolerance to bicarbonate addition (~9 mM) above which both parameters decreased. Nonetheless, the transient highest cellular lipid and TAG contents were obtained at 18 mM bicarbonate, immediately after combined alkaline pH stress and nitrate depletion (day nine), while oil body and TAG accumulation were highly repressed with low carbon supply (2 mM). Despite decreases in the proportions of EPA and DHA, maximum volumetric and cellular EPA and DHA contents were obtained at this stage due to accumulation of TAG containing EPA/DHA. TAG accounted for 74% of the total fatty acid per cell, containing 55% and 67% of the overall cellular EPA and DHA contents, respectively. These results clearly demonstrate that inorganic carbon availability and elevated pH represent two limiting factors for lipid and TAG accumulation, as well as *n*-3 LC-PUFA partitioning into TAG, under nutrient-depleted *P. lutheri* cultures.

## 1. Introduction

Microalgae constitute a source of bioactive compounds offering a variety of nutraceutical and pharmaceutical applications [1,2]. Amongst them, the omega-3 long-chain polyunsaturated fatty acids (*n*-3 LC-PUFA), such as eicosapentaenoic (EPA, 20:5 *n*-3), and docosahexaenoic (DHA, 22:6 *n*-3) acids, are known for their beneficial effects on human health [3,4]. Today, *n*-3 LC-PUFA from marine organisms used in human nutrition are mainly obtained from marine fish oils. While microalgae synthesize *n*-3 LC-PUFA, fish usually obtain EPA via bioaccumulation through the food chain, which increases the susceptibility to be contaminated by pollutants such as heavy metals [2]. The unpleasant odor of the extracted oil and depletion of fish resources [2,5] have also lead to a search for alternative natural resources to meet the growing demand for vegetarian *n*-3 LC-PUFA. Although *n*-3 LC-PUFA production from autotrophic algae is technically possible, several challenges remain before it will be economically feasible [6,7].

In photoautotrophic eukaryotic microalgae, LC-PUFA are mainly accumulated in complex polar lipids (*i.e.*, glycolipids and phospholipids) constituting the membranes, while triacylglycerols (TAG) are predominantly constructed of saturated (SFA) and monounsaturated (MUFA) fatty acids [8,9,10,11]. To produce LC-PUFA-enriched oil, the form most desirable for dietary intake, a better understanding of the mechanisms by which some species are able to incorporate LC-PUFA into storage lipids is required [8,12]. As an example, the freshwater chlorophyte *Parietochloris incisa* constitutes a rare case of an autotrophic alga that is able to accumulate substantial amounts of TAG containing *n*-6 LC-PUFA (arachidonic acid, 20:4 *n*-6, ARA) [13,14]. LC-PUFA partitioning to TAG has been reported to occur also, to a lesser extent, in a few other species (e.g., *Pavlova lutheri*, *Nannochloropsis oculata*, *Thalassiosira pseudonana*, and *Phaeodactylum tricornutum*) [8,15]. The processes by which *n*-3 fatty acids, such as EPA and DHA, are incorporated into TAG are not fully understood but are thought to be species-specific and dependent on growth phases [8]; however, the factors controlling such mechanisms require further investigation.

Nutrient, medium pH, and carbon supplies are three major factors impacting growth and lipid metabolism of microalgae. Lipid synthesis and fatty acid profiles are particularly affected by nutrient availability, and changes are mainly caused by nitrogen, phosphate, sulphate, or silica limitation, occurring with culture age [16,17,18]. Indeed, many microalgae have the ability to produce substantial amounts (*i.e.*, 20%–50% dry cell weight) of TAG under stress conditions, such as high light, alkaline pH, and nutrient depletion, reaching up to ~80% in some species [19,20]. However, most commonly, nutrient starvation induced cessation of cell-growth and accumulation of storage lipid (TAG), containing mostly SFA and MUFA, rather than PUFA [6]. Therefore, the percentage of PUFA relative to total fatty acids in the biomass, such as EPA, usually decrease under nutrient limitation [17,21,22]. Maximal PUFA levels are observed during the early stationary phase, and decrease throughout the stationary phase, with parallel increases in the proportions of SFA and MUFA, such as 14:0, 16:0, and 16:1 fatty acids [18,23,24]. Although it is well-known that nutrient limitation can increase lipid content and general trends in fatty acid profiles are recognized, responses are mostly species-specific [25,26,27]. *P. lutheri* is one of the few species, reported so far, where EPA and DHA levels increased in both the total and TAG fatty acid extracts on the transition to the stationary phase [10].

An adequate supply of inorganic carbon is also essential to maintain photosynthetic, carbon fixation and, hence, growth in photoautotrophic microalgae. Microalgae that grow photoautotrophically use inorganic carbon sources to synthesize, *de novo*, their own organic carbon compounds [28] and inorganic carbon availability represents a limiting factor in algal production. Cultures, therefore, require a supplementation, which is generally achieved using CO_2_-enriched air. In this context, most studies have focused on the effect of gaseous CO_2_ addition on growth, lipid production, and biochemical composition in microalgal cultures [29,30,31,32,33]. The presence of high levels of CO_2_ has been shown to promote the production of biomass and lipids in microalgal cells [33,34]. However, due to the low solubility and low retention times of CO_2_ in the culture media [35], several authors recently investigated the effects of sodium bicarbonate as an inorganic carbon source to stimulate growth and TAG accumulation [36,37,38,39,40,41]. Their results clearly indicate that sodium bicarbonate addition is a promising strategy to increase biomass and oil accumulation in some microalgae. For example, the effect of bicarbonate addition has been demonstrated to be an effective lipid accumulation trigger on two distinct species of algae: the green alga *Scenedesmus* sp. strain WC-1, and the marine diatom *P. tricornutum* [37]. Indeed, bicarbonate addition has been used to increase pH and total dissolved inorganic carbon near the time of nitrate depletion. Independently of carbon levels, high pH stress also resulted in TAG accumulation in different species of Chlorophyta, and, particularly, during nitrogen limitation [42,43]. Nonetheless, it is still unclear whether nutrient depletion can induce lipid and TAG accumulation under a low carbon supply. Peng *et al.* (2013) showed that cell growth and accumulation of cellular lipids in *P. tricornutum* CCMP632 appeared to rely entirely on the fixation of external inorganic carbon under nutrient-stress, with no changes under carbon limitation but significant increases under carbon sufficiency [41]. Moreover, levels of TAG were still synthesized and accumulated under inorganic carbon limitation, coinciding with a cessation in cell growth and cellular lipid accumulation [41].

Aside from the factors triggering lipid accumulation described previously, several studies report the importance of harvesting time to achieve maximum lipid content during batch cultivation [38,39,44]. For example, Jiang *et al.* (2012) studied the variation on lipid content in response to nitrogen limitation in *Dunaliella tertiolecta* and *T. pseudonana*, reporting maximum lipid content on day 10, followed by decline [45]. Cellular lipid content of *Nannochloropsis salina*, re-suspended in nitrate-deplete media and supplement with 2 g L^−1^ bicarbonate, also reached a maximum after 15 days, which was followed by a decrease [39].

Prior to considering the production of *n*-3 LC-PUFA-enriched oils from autotrophic microalgae, it is necessary to identify, and better understand, the mechanisms by which biochemical and environmental factors trigger oil accumulation and LC-PUFA partitioning into TAG with reduced influence on growth. Due to its ability to partition *n*-3 LC-PUFA into TAG [10,12,46], *P. lutheri* (an EPA/DHA-rich marine haptophyte) represents a good candidate for further investigation into the processes responsible for the incorporation of LC-PUFA into storage oils. Our choice was also based on the fact that *P. lutheri*, as with other microalgal species, has been described to be able to incorporate [14C] bicarbonate and to synthesize lipids from this substrate [47,48]. In autotrophic microalgae, as in plants, inorganic carbon can be fixed by RuBisCO via the Calvin-Benson cycle and also by β-carboxylation, using different enzymes, such as phosphoenol pyruvate carboxylase (PEPC), phosphoenol pyruvate carboxykinase (PEPCK), and pyruvate carboxylase [49,50]. Bicarbonate (HCO_3_^−^) can be fixed, glyceraldehyde-3-phosphate (G3P) formed, and this is then used to synthesize organic molecules, such as lipids and carbohydrates [47,51]. Moreover, bicarbonate uptake can be partially explained by active mechanisms of CO_2_ accumulation, such as carbonic anhydrase, which some microalgae have developed alongside RuBisCO activity in order to facilitate the conversion of HCO_3_^−^ to CO_2_ [51,52].

In this context, the work presented here details the associated effects of sodium bicarbonate addition and nitrate limitation on cell growth, lipid body formation, total and TAG fatty acid composition; additionally, several parameters (*i.e.*, volumetric and cellular total fatty acid, TAG, EPA and DHA contents) were used to estimate EPA/DHA-enriched oil accumulation and productivity during batch cultivation of *P. lutheri* under continuous light.

## 2. Results and Discussion

### 2.1. Bicarbonate Addition Promotes Growth, Nitrate Uptake and Lipid Production during Batch Cultivation of *P. lutheri*

**Figure 1:**
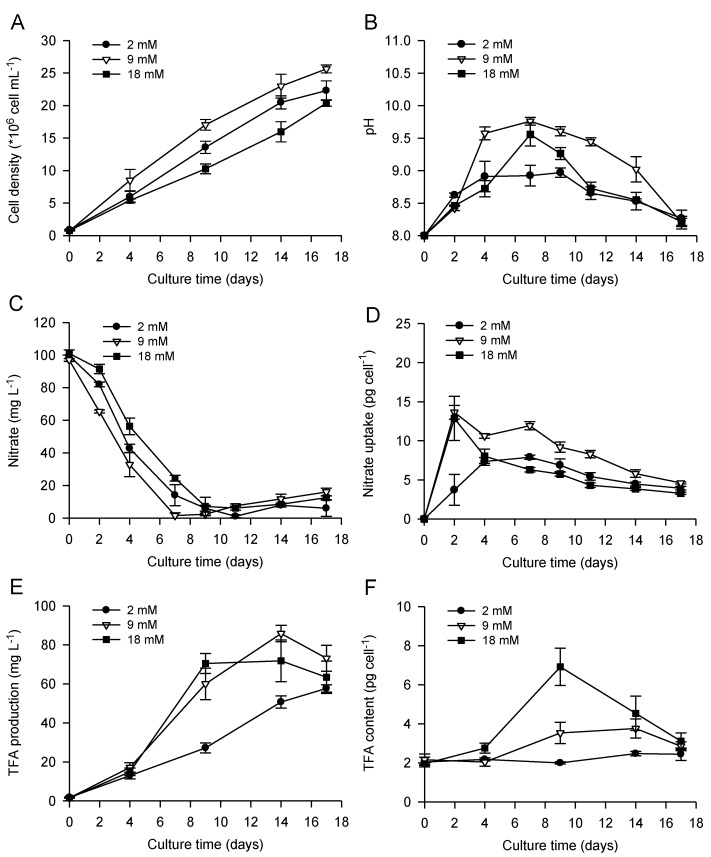
Cell growth (**A**), pH (**B**) and nitrate concentration (**C**) in the culture media, nitrate uptake per cell (**D**), total volumetric and cellular (**E** and **F**, respectively) lipid contents (based on total fatty acids) of *P. lutheri* during batch cultivation supplemented with different initial bicarbonate concentrations. Results are expressed as the mean ± SD of three replicates (*n* = 3).

Bicarbonate addition, and associated alkaline pH stress, has been shown to promote lipid accumulation in a number of microalgae but with species-specific responses with respect to cell division [36,37,38,39,40,42,43]. Gardner *et al.* (2012) demonstrated that bicarbonate addition stopped cell division in the chlorophyte *Scenedesmus* sp., but not in the diatom *P. tricornutum* where the cell cycle could be completed [37]. Similarly, growth of *N. salina* was reduced by bicarbonate addition, whereas no significant effect was reported on growth of *Tetraselmis suecica* [39]. In our studies, nitrate concentration was monitored in the media during batch cultivation of *P. lutheri* to provide an indication of nutrient status and to establish whether there was a correlation between nitrate depletion, growth, and cellular lipid accumulation (Figure 1). The highest growth rates and final cell densities obtained using an initial bicarbonate concentration of 9 mM were associated with a faster nitrate uptake, reaching complete depletion on day seven (Figure 1A,C,D). Growth of *P. lutheri* using 18 mM bicarbonate exhibited a decrease (~20% in comparison to 9 mM bicarbonate) in final cell density associated with a slight delay in the culture to reach complete nitrate depletion. Results indicate that growth and nitrate uptake of *P. lutheri* had an optimum and threshold tolerance to bicarbonate addition (~9 mM) above which a decrease in both parameters was recorded. When supplementing cultures with gaseous CO_2_, Carvalho and Malcata (2005) also described a sensitivity of *P. lutheri* to inorganic carbon supply with increases in biomass up to a concentration of 1% CO_2_ in air (v/v), followed by a decrease at higher concentrations [32].

**Figure 2:**
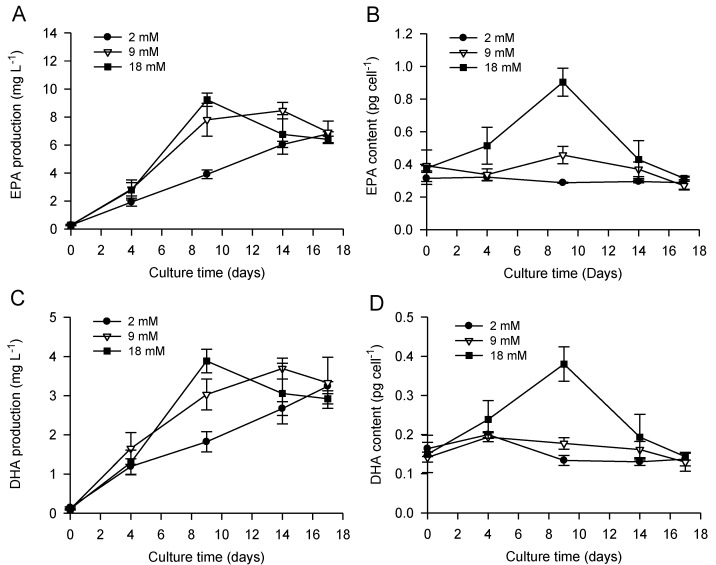
Contents of volumetric and cellular EPA (**A** and **B**, respectively) and DHA (**C** and **D**, respectively) in *P. lutheri* during batch cultivation supplemented with different initial bicarbonate concentrations. Results are expressed as the mean ± SD of three replicates (*n* = 3).

The addition of bicarbonate also had significant effects on the alkalinity of the cultures reaching maximum pH levels of pH 9.6 and 9.8 after nine days, applying 18 and 9 mM bicarbonate, respectively (Figure 2B). However, a rise in pH seemed to be associated with cell division and faster growth using 9 mM bicarbonate, suggesting increased carbon fixation, which was consistent with results obtained regarding nitrate uptake. Indeed, CO_2_ uptake during growth of photosynthetic microalgae has previously been reported to lead to an increase in pH and a decrease in CO_2_ partial pressure, with CO_2_ replacement occurring more slowly than consumption [39]. At these elevated pH levels, a slight precipitation of salts in the culture was observed, using 9 and 18 mM bicarbonate, probably due to a shift in the inorganic form of carbon to carbonate (CO_3_^2−^) and insoluble carbonates of metals formation, which are not readily utilized by photosynthetic marine algae and can reduce growth and photosynthesis in some species [53]. In *P. lutheri*, no complete cessation of cell growth was observed with pH rise induced by bicarbonate addition, but a decrease in final cell density with 18 mM bicarbonate, as mentioned previously. Due to the observed salt precipitation, all results in this study are expressed per cell, and not per dry weight as in other studies.

After full nitrate depletion, the maximum cellular lipid content (based on total fatty acids, Figure 1F) was higher in cultures supplemented with 18 mM bicarbonate (6.9 ± 1.0 pg cell^−1^), compared to cultures with 9 mM bicarbonate addition (3.8 ± 0.5 pg cell^−1^). Despite the precipitation of salts observed, this result suggests higher inorganic carbon availability with increased bicarbonate supply for *de novo* synthesis of organic carbon compounds. Interestingly, lipid accumulation was highly repressed at low carbon supply (2 mM bicarbonate), showing almost no changes over time, even under nutrient depletion. As observed in previous studies for several chlorophyte species [39,43], cellular lipid accumulation in *P. lutheri* also correlated with elevated pH at the time of nitrate depletion. Despite the proven ability of bicarbonate addition to enhance cellular lipid content in *P. lutheri*, carbon supply seems to be a limiting factor for lipid accumulation. Indeed, Toledos-Cervantes *et al.* (2013) also reported that lipid accumulation in *Scenedesmus obtusiusculus* significantly increased at 5% CO_2_, but not when the species was batch-cultivated at 0.04% CO_2_ (air without CO_2_-enrichment) [44]. In *P. lutheri*, Tonon *et al.* (2002) showed a decrease in the total fatty acid content per cell upon entry into the stationary phase [10], which could be explained by limited carbon availability using aeration by shaking the flasks at 150 rpm (without additional carbon supply).

Moreover, as suggested in some previous studies [39,44,45], lipid accumulation is a transient metabolic process reaching a maximum shortly after nutrient limitation. Cellular lipid content in *P. lutheri* reached a maximum after nine and 14 days, using 18 and 9 mM bicarbonate, respectively, and subsequent decline was observed, indicating that lipid accumulation ceased and/or cellular lipids were being utilized or degraded. Interestingly, lower in cellular lipid content corresponded to a reduction in pH in the culture media after nine days. During batch culture, harvesting time constitutes, therefore, a critical parameter to obtain the highest cellular lipid content and achieve optimum productivity.

Finally, maximum volumetric lipid production (based on total fatty acids, Figure 1E) was observed after 14 days, using 9–18 mM bicarbonate addition (~70–85 mg L^−1^).

### 2.2. Combined Effect of Nitrogen Limitation and Bicarbonate Addition on Total Fatty Acid Composition and *n*-3 LC-PUFA Production

It is well established that lipid metabolism and fatty acid profiles are highly dependent on nutrient availability [16,17,18]. Nitrate limitation or starvation are known key factors controlling oil accumulation and associated changes in fatty acid composition [22,27,54,55]. In *P. lutheri*, independent of the bicarbonate concentrations, the main alterations in total fatty acid (TFA) profiles due to nitrate limitation were accounted for by relative increases in SFA and MUFA levels, especially 16:0 and 16:1 *n*-7, concomitant with a decrease in the proportion of PUFA (*i.e.*, 18:4 *n*-3, EPA and DHA). 16:0 and 16:1 *n*-7 fatty acids increased from 18.5%–19.7% and 17.9%–24.6% to 26.2%–29.9% and 29.3%–30.3% of TFA, respectively; while EPA and DHA decreased from 16.1%–18.7% and 8.6%–9.6% to 9.4%–11.6% and 4.2%–5.3% of TFA, respectively (Table 1). These results are in agreement with several studies showing that in most species, levels of PUFA such as EPA decreased under nutrient limitation with parallel increases in the proportions of SFA and/or MUFA [17,18,22,23,24]. This can be explained by the fact that nitrate starvation induces storage lipid (TAG) accumulation, which contains mainly SFA and MUFA rather than PUFA [6]. Moreover, changes in fatty acid composition in algae are often related to the proportions of the different lipid classes, which have distinctive fatty acid profiles [36,56,57]. Breuer *et al.* (2012) suggested that the accumulation of TAG with a different fatty acid composition than that of functional and structural lipids in the oleaginous strains, or a shift in lipid class composition (e.g., reduction in thylakoid membrane content) in non-oleaginous strains, could explain these variations upon nitrogen starvation [20]. In order to highlight this specific issue, further details will be provided in the following sections (*cf.* Section 2.3 and Section 2.4) regarding the combined effect of bicarbonate addition and nitrate limitation on TAG accumulation and TAG fatty acid composition of *P. lutheri*. Surprisingly, during batch cultivation of *P. lutheri* under a relative high light (240 µM photons m^−2^ s^−1^), EPA and DHA levels were increasing after nitrate depletion in both total and TAG fatty acid extracts [10]. Previously, the percentage of EPA and DHA in neutral lipids of *P. lutheri* have been reported to increase with light intensity [36], highlighting the importance of factors such as light and temperature associated to those promoting TAG accumulation on LC-PUFA synthesis and partitioning into lipid classes. pH increases with culture age, especially with bicarbonate addition, may also contribute to the fatty acid changes observed in *P. lutheri* after nitrate depletion.

**Table 1:** Total fatty acid profile and content in batch culture of *P. lutheri* supplemented with different initial bicarbonate concentrations.

	Bicarbonate (mM)					
	Before *N*-Limitation			After *N*-Limitation		
Fatty Acids (% TFA)	2	9	18	2	9	18
*Saturated fatty acids*						
14:0	17.3 ± 0.7	15.2 ± 0.7	15.5 ± 2.8	14.3 ± 0.3	13.8 ± 0.3	14.7 ± 1.3
16:0	18.5 ± 1.1	19.7 ± 0.3	19.0 ± 2.0	26.2 ± 0.1	27.4 ± 0.8	29.9 ± 1.9
18:0	2.1 ± 1.8	1.9 ± 0.2	2.7 ± 2.5	0.6 ± 0.0	1.5 ± 0.3	0.7 ± 0.2
Sum of SFA	37.8 ± 0.4	36.9 ± 1.0	37.2 ± 7.2	41.1 ± 0.2	42.6 ± 0.2	45.3 ± 0.6
*Monounsaturated fatty acids*						
16:1 *n*-7	24.6 ± 1.0	20.2 ± 1.3	17.9 ± 0.5	30.3 ± 0.1	29.4 ± 0.6	29.3 ± 1.6
18:1 *n*-7	1.3 ± 0.2	1.0 ± 0.1	1.3 ± 0.1	1.5 ± 0.1	2.2 ± 0.2	1.4 ± 0.1
18:1 *n*-9	2.0 ± 0.1	2.6 ± 0.6	2.0 ± 0.1	2.1 ± 0.1	2.9 ± 0.2	1.5 ± 0.2
Sum of MUFA	27.8 ± 0.9	23.9 ± 1.4	21.2 ± 0.3	33.9 ± 0.3	34.4 ± 0.8	32.2 ± 1.4
*Polyunsaturated fatty acids*						
18:2 *n*-6	0.8 ± 0.1	1.2 ± 0.2	1.4 ± 0.3	1.6 ± 0.1	2.0 ± 0.1	1.9 ± 0.2
18:3 *n*-6	tr.	0.6 ± 0.1	tr.	tr.	0.3 ± 0.0	tr.
18:3 *n*-3	1.1 ± 0.3	1.1 ± 0.2	0.4 ± 0.1	0.7 ± 0.0	0.8 ± 0.1	0.6 ± 0.0
18:4 *n*-3	4.0 ± 0.5	5.7 ± 0.1	7.2 ± 1.4	2.0 ± 0.0	2.0 ± 0.2	2.6 ± 0.1
20:4 *n*-6	0.8 ± 0.1	0.6 ± 0.3	0.9 ± 0.2	0.9 ± 0.3	1.0 ± 0.1	0.8 ± 0.2
20:5 *n*-3	16.1 ± 0.2	16.5 ± 0.4	18.7 ± 4.1	11.6 ± 0.2	9.8 ± 0.6	9.4 ± 1.0
22:5 *n*-3	0.8 ± 0.3	0.9 ± 0.2	0.6 ± 0.0	1.2 ± 0.1	1.1 ± 0.1	1.5 ± 0.3
22:6 *n*-3	9.0 ± 0.3	9.6 ± 1.4	8.6 ± 1.5	5.3 ± 0.1	4.3 ± 0.3	4.2 ± 0.4
Sum of PUFA	32.3 ± 0.5	36.1 ± 1.4	37.9 ± 6.9	23.4 ± 0.5	21.3 ± 1.1	21.0 ± 1.6
Others	2.0 ± 0.4	3.2 ± 0.3	3.7 ± 0.6	1.6 ± 0.1	1.5 ± 0.1	1.5 ± 0.1
*n*-3	29.9 ± 0.8	33.8 ± 1.5	35.5 ± 7.1	20.1 ± 0.3	18.1 ± 1.1	18.3 ± 1.2
*n*-6	2.4 ± 0.4	2.3 ± 0.2	2.3 ± 0.1	3.3 ± 0.3	3.3 ± 0.1	2.7 ± 0.3
TFA (pg cell^−1^)	2.2 ± 0.1	2.0 ± 0.2	2.8 ± 0.3	2.5 ± 0.1	3.8 ± 0.5	6.9 ± 1.0
TFA (mg L^−1^)	13.0 ± 1.7	17.2 ± 2.5	14.9 ± 1.9	57.7 ± 1.9	85.8 ± 4.2	71.9 ± 10.7

Results are expressed as the mean ± SD of three replicates (*n* = 3). tr., traces; MUFA, monounsaturated fatty acids; PUFA, polyunsaturated fatty acids; SFA, saturated fatty acids; TFA, total fatty acids.

On the other hand, the observed changes in fatty acid composition (Table 1) that occurred with increased bicarbonate concentrations were only small. Prior to nitrate limitation (day four), an increase in PUFA, mainly 18:4 *n*-3 (from 4.0% to 7.2% of TFA), was observed in association with a decrease in MUFA, mainly 16:1 *n*-7 (from 24.6% to 17.9%). While only the proportion of SFA (especially 16:0, from 26.2% to 29.9%) increased, slight decreases in EPA and DHA levels were observed after nitrate limitation. Indeed, Carvalho and Malcata (2005) previously suggested that increased levels of CO_2_ as inorganic carbon favored the total lipid content, but decreased the amounts of PUFA in *P. lutheri* [32].

Despite the observed decreases in the proportions of EPA and DHA (% of TFA) under nitrate limitation, maximum volumetric and cellular EPA and DHA contents (Figure 2) were obtained more quickly using an initial bicarbonate concentration of 18 mM immediately after nitrate depletion (day nine). Therefore, the highest volumetric EPA and DHA productivity reached 9.2 ± 0.5 and 3.9 ± 0.3 mg L^−1^, respectively; and maximum cellular EPA and DHA contents attained 0.9 ± 0.1 and 0.4 ± 0.1 pg cell^−1^, respectively. Our values are similar to those developed in order to improve the production and the storage of EPA/DHA during *P. lutheri* batch cultivation [58]. At the lowest temperature (15 °C) and light intensity (50 µM photons m^−2^ s^−1^), growth rate during the exponential growth phase was multiplied by 1.5, biomass at the end of the culture was similarly increased and the maximum cellular EPA and DHA contents reached approximately 0.80 and 0.45 pg cell^−1^, respectively [58]. In our study, *P. lutheri* cells were cultivated at 20 °C and 100 µM photons m^−2^ s^−1^; the relatively high cellular EPA and DHA contents might therefore be attributed to the cellular lipid accumulation triggered by bicarbonate addition described previously and not to light and temperature.

### 2.3. Increased TAG Accumulation and Oil-Body Formation Is to a Large Extent Attributable to Increased Carbon Availability

**Figure 3:**
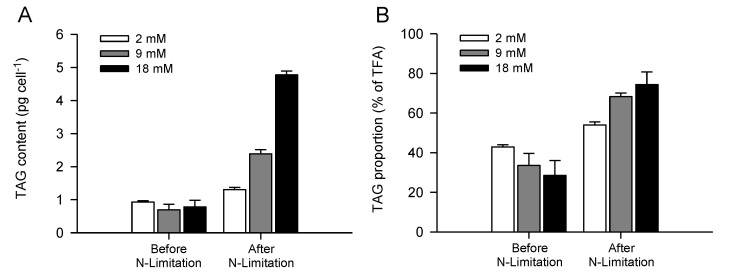
Maximum cellular TAG content (**A**) and overall proportion of TAG (**B**) before and after nitrogen limitation of batch-cultivated *P. lutheri* supplemented with different initial bicarbonate concentrations. Results are expressed as the mean ± SD of two replicates (*n* = 2).

Several studies have recently suggested that carbon availability is a key metabolic factor controlling oil biosynthesis and carbon partitioning between starch and oil in different microalgal species [40,59,60]. Using the green microalga *Chlamydomonas reinhardtii*, which is able to grow mixotrophically or autotrophically, as a model system, acetate (organic carbon) and bicarbonate (inorganic carbon) have been described as limiting factors and central molecules in lipid droplet synthesis under nutrient limitation [38,59,60]. In the present study, similar results have been obtained for the first time for *P. lutheri*, where TAG accumulation, expressed as cellular TAG content, always occurred close to nitrate depletion and was stimulated by bicarbonate addition, reaching 4.8 ± 0.1 pg cell^−1^ using 18 mM bicarbonate (Figure 3A). In the diatom *P. tricornutum* and several chlorophyte species grown autotrophically, neutral lipid accumulation (*i.e.*, volumetric and specific Nile Red fluorescence intensity) associated with bicarbonate was dependent on concentration and time of addition [37,38,40]. The overall proportion of TAG, expressed in percentage of TFA, increased from 29%–42% before nitrate limitation to 54%–74% after nitrate depletion (Figure 3B), irrespective of bicarbonate concentration. After nitrate limitation, bicarbonate induced TAG accumulation mechanisms in *P. lutheri* was also highlighted by a higher overall proportion of TAG obtained with 9 and 18 mM (68% and 74%, respectively) in comparison to low bicarbonate supply (2 mM, 54% of TFA). Previously, neutral lipids and glycolipids have been described as the major constituents in *P. lutheri* cells grown at 16 °C, under 100 µM photons m^−2^ s^−1^, using 2.07 mM bicarbonate and harvested in the mid-exponential growth phase, accounting for ~36% and ~54% of the total lipids, respectively [36]. The relatively high proportion of TAG observed in *P. lutheri* after nitrate depletion could therefore be explained by a membrane re-cycling process, converting some existing membrane polar lipids into TAG, as suggested by Hu *et al.* (2008) [19].

Additionally, our results showed that highest TAG accumulation occurred at elevated pH in the media after nitrate depletion (day nine), using both 9 and 18 mM bicarbonate (data not shown), as described for maximum cellular lipid content obtained in *P. lutheri* (*cf.* Section 2.1), and several other previous studies on microalgae using Nile Red fluorescence to quantify TAG accumulation [39,42,43].

**Figure 4:**
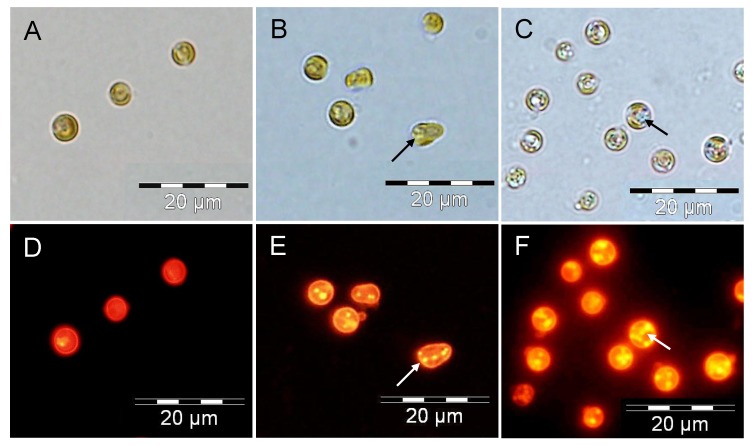
Oil body/droplet formation in nitrate-depleted cells of *P. lutheri* batch-cultivated in the presence of different initial bicarbonate concentrations. Neutral lipid accumulation in lipid bodies was visualized in algal cells with the fluorescent dye Nile Red. Cells grown in the presence of 2, 9, and 18 mM bicarbonate (**A**–**D**, **B**–**E** and **C**–**F**, respectively). The arrows indicate lipid bodies.

Nile Red fluorescence has been correlated to cellular TAG concentrations [61,62,63]. This method has been developed, and is now intensively used, for a rapid and quantitative measurement of neutral lipids [37,38,39], as well as the visualization of lipid body formation in different microalgal species [54,59,60]. As expected, in *P. lutheri*, oil droplets revealed by Nile Red staining were much smaller and less abundant in nitrate-depleted cells in the presence of 9 mM bicarbonate compared to cells grown in the presence of 18 mM bicarbonate (Figure 4). By contrast, oil bodies were hardly visible in *P. lutheri* cells grown at an initial bicarbonate concentration of 2 mM, confirming the inability of this species to accumulate TAG when under low inorganic carbon concentrations.

### 2.4. Increasing Inorganic Carbon Supply Enhances Accumulation of TAG Containing *n*-3 LC-PUFA

Changes in total fatty acid content and composition induced by nutrient limitation have been studied in details for various species (*cf.* Section 2.2). However, the effect of nutrient depletion on TAG fatty acid composition and LC-PUFA partitioning into TAG has been less well described and deserves further investigation.

In studies reporting on the capability of some species to partition LC-PUFA into TAG after nutrient limitation [58], cellular EPA and DHA content in TAG are often provided without detailed TAG fatty acid composition (% TFA). In *P. tricornutum*, TAG fatty acid composition under nitrate starvation showed a slight increase in 16:0 fatty acids accompanied by a decrease in 16:1 *n*-7, but without any significant changes in EPA level (accounting for ~5% of TFA) [54]. In the freshwater species *Monodus subterraneus* (Eustigmatophyta), the main changes in TAG fatty acid composition as a result of phosphate starvation consisted of an increase in the proportion of 16:1, combined with a decrease in 18:1 *n*-9 and EPA [17]. *P. incisa* has so far been described as the only autotrophic microalga able to incorporate ARA into TAG, reaching up to 47% in the stationary phase and exceeding 60% of TFA under nitrogen starvation [13,14]. Regarding *P. lutheri* in this study (Table 2), similar to changes in total fatty acid composition, the main variations observed were an increase in 16:0 and 16:1 *n*-7 correlated with a small decrease in EPA level after nitrate depletion. Additionally, C18 fatty acids (e.g., 18:0, 18:1 *n*-9, 18:1 *n*-7, and 18:2 *n*-6) slightly decreased after nitrate depletion. Changes in TAG fatty acid composition of *P. lutheri* were also observed with increased bicarbonate concentrations, with a strongest effect detected after nitrate limitation (Table 2). The proportion of MUFA, mainly 16:1 *n*-7, decreased significantly, while the level of *n*-3 LC-PUFA, especially EPA and DHA, increased with bicarbonate addition. Highest percentages of EPA (~10%) and DHA (~4%) partitioned to TAG were obtained using 9–18 mM bicarbonate before and after nitrate limitation, respectively.

**Table 2:** TAG fatty acid profile and content in batch culture of *P. lutheri* supplemented with different initial bicarbonate concentrations.

	Bicarbonate (mM)					
	Before *N*-Limitation			After *N*-Limitation		
Fatty Acids (% TFA)	2	9	18	2	9	18
*Saturated fatty acids*						
14:0	11.2 ± 0.8	9.6 ± 0.9	9.6 ± 0.9	10.7 ± 0.6	9.7 ± 0.2	10.3 ± 0.4
16:0	22.6 ± 2.1	22.4 ± 1.3	22.4 ± 1.3	30.7 ± 0.8	27.4 ± 0.3	29.5 ± 0.5
18:0	5.6 ± 1.4	5.5 ± 1.7	5.5 ± 1.7	0.8 ± 0.1	0.9 ± 1.2	0.9 ± 0.2
Sum of SFA	39.5 ± 0.1	37.5 ± 3.9	37.5 ± 3.9	42.2 ± 1.4	38.1 ± 0.3	40.7 ± 1.4
*Monounsaturated fatty acids*						
16:1 *n*-7	22.0 ± 3.3	22.9 ± 10.3	15.9 ± 0.3	36.5 ± 0.2	33.9 ± 0.1	30.8 ± 0.5
18:1 *n*-7	3.0 ± 0.9	4.5 ± 2.2	3.7 ± 0.5	2.0 ± 0.0	1.7 ± 0.1	1.8 ± 0.3
18:1 *n*-9	5.6 ± 2.1	4.5 ± 1.1	5.4 ± 0.8	2.1 ± 0.1	3.3 ± 0.2	2.4 ± 0.0
Sum of MUFA	30.7 ± 2.1	31.8 ± 7.1	24.9 ± 0.6	40.7 ± 0.2	38.9 ± 0.2	35.0 ± 0.2
*Polyunsaturated fatty acids*						
18:2 *n*-6	3.5 ± 0.3	3.4 ± 0.3	3.9 ± 0.3	0.4 ± 0.1	0.4 ± 0.0	0.3 ± 0.0
18:3 *n*-6	0.2 ± 0.0	0.4 ± 0.0	0.8 ± 0.4	0.4 ± 0.0	0.6 ± 0.1	0.4 ± 0.1
18:3 *n*-3	1.2 ± 0.2	1.7 ± 0.2	2.0 ± 1.2	2.0 ± 0.0	2.4 ± 0.1	3.0 ± 0.2
18:4 *n*-3	1.5 ± 0.4	2.1 ± 0.3	1.7 ± 0.0	1.0 ± 0.1	1.5 ± 0.1	1.5 ± 0.1
20:4 *n*-6	1.3 ± 0.3	0.9 ± 0.2	1.6 ± 0.3	1.0 ± 0.1	1.1 ± 0.1	1.3 ± 0.1
20:5 *n*-3	7.6 ± 0.6	10.1 ± 1.4	10.8 ± 0.4	5.9 ± 0.5	7.9 ± 0.1	8.2 ± 0.5
22:5 *n*-3	0.4 ± 0.0	0.6 ± 0.0	0.7 ± 0.1	0.7 ± 0.1	1.1 ± 0.2	1.5 ± 0.1
22:6 *n*-3	3.5 ± 0.8	2.9 ± 0.1	1.4 ± 0.4	2.5 ± 0.5	4.2 ± 0.4	4.2 ± 0.3
Sum of PUFA	19.3 ± 1.4	22.1 ± 2.2	23.0 ± 1.6	13.9 ± 1.3	19.2 ± 0.6	20.6 ± 1.2
Others	10.6 ± 3.5	8.6 ± 1.0	16.9 ± 3.8	3.3 ± 0.3	3.8 ± 0.1	3.8 ± 0.1
*n*-3	14.3 ± 1.9	17.5 ± 1.8	16.7 ± 1.8	10.4 ± 1.2	15.3 ± 0.8	15.9 ± 0.9
*n*-6	5.0 ± 0.6	4.6 ± 0.4	6.3 ± 0.2	3.4 ± 0.1	3.9 ± 0.2	4.6 ± 0.3
TAG (pg cell^−1^)	0.9 ± 0.1	0.7 ± 0.2	0.8 ± 0.2	1.3 ± 0.1	2.4 ± 0.1	4.8 ± 0.1
TAG (% TFA)	42.9 ± 1.1	33.6 ± 6.1	28.5 ± 7.6	54.0 ± 1.6	68.3 ± 1.9	74.4 ± 6.4

Results are expressed as the mean ± SD of two replicates (*n* = 2). MUFA, monounsaturated fatty acids; PUFA, polyunsaturated fatty acids; SFA, saturated fatty acids; TFA, total fatty acids.

**Figure 5:**
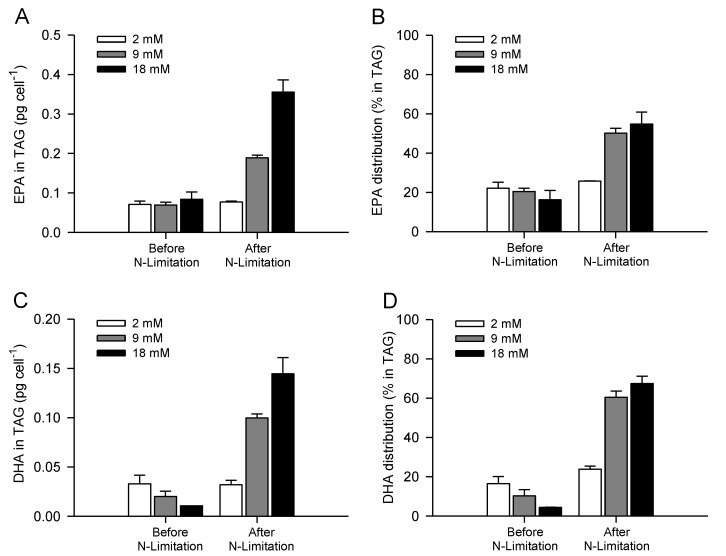
TAG cellular EPA and DHA (**A** and **C**, respectively) contents and overall distribution into TAG (**B** and **D**, respectively) before and after nitrogen limitation of batch-cultivated *P. lutheri* supplemented with different initial bicarbonate concentrations. Results are expressed as the mean ± SD of two replicates (*n* = 2).

Furthermore, Guckert and Cooksey (1990) also suggested that cells inhibited by stress (*i.e.*, alkaline pH) showed an increase in TAG accumulation but a decrease in both membrane lipid classes with unstable membrane lipid fatty acid profiles similar to the TAG, *i.e.*, less unsaturated [42]. pH rise with culture age could, therefore, also have contributed to the small decrease in EPA levels observed in TAG of *P. lutheri* after nitrate depletion. At bicarbonate concentrations of 18 mM, the combined increases in cellular TAG content and *n*-3 LC-PUFA accumulation in TAG lead to significant increases in TAG cellular EPA and DHA contents, reaching a maximum of 0.36 ± 0.03 and 0.14 ± 0.02 pg cell^−1^, respectively (Figure 5A,C). As a consequence, TAG of *P. lutheri* contained 55% and 67% of the overall cellular EPA and DHA contents, respectively (Figure 5B,D). EPA has been previously shown to be concentrated particularly in monogalactosylacylglycerols (MGDG) and TAG in *P. lutheri*; conversely, DHA was dispersed through various classes, especially within TAG, diphosphatidylglycerols (DPG), and betaine lipids [12,46,64].

Bicarbonate supplementation therefore seems to trigger the accumulation of TAG containing *n*-3 LC-PUFA and at a lesser extent *n*-3 LC-PUFA partitioning into TAG in *P. lutheri*, even though some decreases were observed in the proportions of EPA and DHA after nitrate depletion.

## 3. Experimental Section

### 3.1. Strain and Culture Conditions

An axenic strain of *Pavlova lutheri* CCAP 931/6 was obtained from the Culture Collection of Algae and Protozoa [65] at the Scottish Marine Institute (SAMS Research Services Ltd., Oban, UK) and cultivated in the laboratory at the National University of Ireland Galway. *P. lutheri* were cultivated under batch conditions on F/2-RSE medium in 2000 mL glass Erlenmeyer flasks in controlled plant growth chambers with adjustable light cassettes (Binder GmbH, Germany) at 20 °C and under continuous illumination (100 µM photons m^−2^ s^−1^), provided by lumilux cool daylight fluorescent lamps (OSRAM L18W/865, Germany), using a working volume of 1600 mL. F/2-RSE medium, a modified version of Guillard’s (1975) F/2 medium [66] where filtered seawater is substituted by ReefSalt (H2Ocean, Pro+, UK), was composed of 34 g L^−1^ ReefSalt complemented with: 5.65 mg L^−1^ NaH_2_PO_4_·2H_2_O, 100 mg L^−1^ NaNO_3_, 4.16 mg L^−1^ Na_2_EDTA·2H_2_O, 3.15 mg L^−1^ FeCl_3_·6H_2_O, 9.8 μg L^−1^ CuSO_4_·5H_2_O, 22 μg L^−1^ ZnSO_4_·7H_2_O, 10 μg L^−1^ CoCl_2_·6H_2_O, 0.18 mg L^−1^ MnCl_2_·4H_2_O, 6.3 μg L^−1^ Na_2_Mo_4_·2H_2_O, 0.1 mg L^−1^ Thiamine-HCl, 0.5 μg L^−1^ Vitamin B_12_, 0.5 µg L^−1^ Biotin. It is important to note that ReefSalt contains an initial inorganic carbon concentration of ~30 mg L^−1^ [67]. The medium was sterilized by autoclaving for 20 min at 120 °C. Vitamins were filtered and added when the medium cooled to room temperature after autoclaving. Final F/2-RSE medium containing 2.43, 9.28, and 18.21 mM sodium bicarbonate (0.204, 0.78 and 1.53 g L^−1^, respectively) was used to investigate the effects of inorganic carbon supplementation (note: Bicarbonate concentrations indicated in the manuscript have been rounded to 2, 8 and 18 mM, respectively). Bicarbonate powder was added after autoclaving, under the laminar hood to prevent contamination, and then pH adjusted to 8.0 by adding 1 M HCl. Inocula were carried out using daily-diluted cultures with fresh medium in order to maintain the cells in exponentially growing stage before starting the experiment. Cultures were agitated by air-bubbling (~0.03%–0.04% CO_2_). The initial bicarbonate concentration used is closed to 2.07 mM, the usual concentration of bicarbonate added in artificial seawater [36]. Each experiment was performed, using an initial cell density of ~10^6^ cell mL^−1^, over 17 days and carried out with three independent replications for each treatment. Samples were collected on days 4, 9, 14, and 17 to determine growth parameters and for lipid and fatty acid analysis.

### 3.2. Growth Parameters, Medium pH, Biomass Harvesting and Storage

Due to salt precipitation occurring during growth using high initial sodium bicarbonate concentrations (e.g., 9 and 18 mM bicarbonate), the biomass was not estimated by dry weight. Culture growth and all parameters were estimated on the basis of cell density determined using a Neubauer hemocytometer after immobilizing the cells with Lugol 5% and appropriate dilution. Medium pH was measured using a bench CyberScan pH 510 Meter (Eutech Instruments Pte Ltd, Singapore). *P. lutheri* cells were gently harvested by centrifuging (1200 × *g* for 10 min) using a Hettich Rotina 38R centrifuge (Andreas Hettich GmbH, Germany). The pellets obtained were then frozen, and stored at −20 °C prior to analysis.

### 3.3. Nitrate Determination

Nitrate concentration was determined according to the modified method reported by Collos *et al.* (1999) [68]. Standard solutions (from 0 to 100 mg L^−1^) or medium samples collected previously were diluted 10-fold with distilled water and residual nitrate content was directly determined according to optical density measured at 220 nm using a Cary 50 Scan UV-Visible spectrophotometer (Varian, UK).

### 3.4. Nile Red Staining and Microscopy

The Nile Red staining method was used to visualize the intracellular lipid bodies as an indicator of TAG formation [69]. Aliquots (200 µL) of *P. lutheri* cultures were stained with 2 µL of Nile Red (0.5 mg mL^−1^ in dimethylsulfoxide), incubated at room temperature for 5 min, and immediately observed by fluorescence microscopy (Olympus BX51, UK).

### 3.5. Lipid Extraction and Analysis

All chemicals used in the experiment were of analytical grade, and were purchased from Fischer Scientific (Leicestershire, LE11 5RG, UK). Total lipids of *P. lutheri* cells were extracted using ultrasounds (twice, 15 and 30 min, respectively) in a chloroform/methanol/water (2/2/1, v/v/v) system according to Bligh and Dyer (1959) [70]. After phase separation, the chloroform layer, containing the lipids, was collected, and two additional extractions were carried out by adding each time 2 mL of chloroform to the remaining methanol/water phase. The solvents were removed by evaporating under high vacuum using a rotary evaporator (Büchi, Switzerland) and all samples were dissolved in a known volume of chloroform. The neutral lipid classes were separated by thin-layer chromatography (TLC) on Silica Gel 60 plates (Merck, Darmstadt, Germany) with hexane/diethyl ether/glacial acetic acid (70:30:1, v/v/v), according to Li *et al.* (2010) [71]. Fish oil (Menhaden Oil, Catalog No.: 47116, Supelco, Bellefonte, PA, USA) was included on each TLC plate as TAG standard. TAG was recovered from the TLC plates for gas chromatographic analysis following a brief exposure to iodine vapour. For charring, plates were sprayed with a solution of 10% (v/v) H_2_SO_4_ in methanol, and heated until spots appeared.

### 3.6. Fatty Acid Analysis

Fatty acid methyl esters (FAME) from total lipids and TAG were obtained by transmethylation of the freeze-dried cells or TAG extracts with dry methanol containing 2% (v/v) H_2_SO_4_ and heating at 80 °C for 1.5 h with continuous stirring under a nitrogen atmosphere [54]. Gas chromatographic analysis of FAME was performed on an Agilent 7890A GC/5975C MSD Series (Agilent Technologies, Santa Clara, CA, USA) equipped with a flame ionization detector and a fused silica capillary column (DB-WAXETR, 0.25 mm × 30 m × 0.25 μm, Agilent Technologies, Catalog No.: 122-7332). Samples were injected in split mode (split ratio 20:1) using an Agilent auto-injector 7683B series. Hydrogen was used as a carrier gas. The injector and detector temperatures were 250 °C and 300 °C, respectively. The temperature was programmed at 140 °C for 1 min, raised from 140 to 200 °C by a rate of 15 °C min^−1^, and then from 200 to 250 °C at a rate of 2 °C min^−1^. Agilent MSD Productivity ChemStation Software (Catalog No.: G1701EA E.02.00, Santa Clara, CA, USA) was used for instrument control, data acquisition and data analysis (integration, retention times and peak areas). Identification of FAME was obtained by co-chromatography with authentic commercially available FAME standards (Supelco™ 37 Component FAME Mix, Catalog No.: 47885-U, Supelco, Bellefonte, PA, USA) and FAME of fish oil (Menhaden Oil, Catalog No.: 47116, Supelco, Bellefonte, PA, USA). Total fatty acid, EPA and DHA contents were quantified by comparison with a known amount of added pentadecanoic acid 15:0 (Pentadecanoic acid, 99%, Catalog No.: A14664-09, Alfa Aesar, Heysham, LA3 2XY, UK) as internal standard.

## 4. Conclusions

The capacity of some algal species to accumulate TAG containing LC-PUFA, particularly during environmental changes, depends on the regulation of various metabolic pathways. In this study, we demonstrated for the first time, to our knowledge, that cellular lipid content, oil body formation and TAG containing *n*-3 LC-PUFA accumulation induced by nitrate depletion rely mainly on inorganic carbon availability in *P. lutheri*. Although growth and nitrate uptake present an optimum and threshold tolerance to bicarbonate addition above which both parameters decreased, carbon supply seems to be one of the limiting factor involved in *n*-3 LC-PUFA-enriched oil production in *P. lutheri*, besides other primordial factors, such as medium pH, light and temperature. Indeed, lipid and TAG accumulation enhanced by bicarbonate addition at the time of nitrate depletion in *P. lutheri* was correlated with pH increase during the growth of photosynthetic microalgae. During batch cultivation, harvesting time constitutes also a critical parameter to achieve the best productivity. As a consequence, although an industrial process would require a light/dark cycle and our results are based on cultures exposed to continuous light, which overrides some of the cell cycle control mechanisms, this study contributes to optimized culture strategies applied to develop *n*-3 LC-PUFA-enriched oil production systems using autotrophic microalgae. Our findings also constitute an important step towards an improved understanding of the mechanisms involved in lipid metabolism regulation, and more specifically oil accumulation and LC-PUFA partitioning in microalgae.

## Abbreviations

DHA: docosahexaenoic acid
EPA: eicosapentaenoic acid
LC-PUFA: long-chain polyunsaturated fatty acids
PUFA: polyunsaturated fatty acids
TAG: triacylglycerols
